# Rethinking the shoe: is CT perfusion the optimal screening tool for acute stroke patients?

**DOI:** 10.1007/s00330-023-10336-5

**Published:** 2023-10-18

**Authors:** Koenraad H. Nieboer

**Affiliations:** Department of Radiology and Medical Imaging, Vrije Universiteit Brussel (VUB), Universitair Ziekenhuis Brussel, Laarbeeklaan 101, 1090, Brussels, Belgium

In the last years, we have seen a dramatic increase in the use of brain perfusion imaging in suspected stroke patients. This increase has taken place since the publication of multiple studies concerning the safety and efficacy of intravenous thrombolysis and mechanical thrombectomy in an early and extended time window after the start of symptoms or in the case of wake-up stroke [1, 2]. As radiologists, we can perform MRI perfusion (MRP) or CT perfusion (CTP) to obtain and estimate the brain infarct’s potential penumbra and ischemic core.

Despite MRP being the most accurate and safe technique in terms of radiation, CTP is more frequently performed in the diagnostic evaluation of an acute stroke patient due to its much higher availability. According to the European Stroke Organization guidelines [2], obtaining perfusion imaging in patients with a witnessed stroke is unnecessary within 4.5 h after the onset of the symptoms. In reality, however, aiming to find all patients eligible for thrombectomy, whether or not in combination with IV thrombolysis, patients presenting with stroke or stroke-like symptoms are systematically referred for perfusion imaging, often with CT.

CTP is a relatively complex examination, requiring sufficient training and experience to ensure reliable data post-processing and interpretation. Lately, fully automated post-processing tools were made available, providing relevant perfusion maps and core/penumbra volumes. These automated tools are convenient, and “flag” the suitable patients for treatment. But sometimes the results are not optimal, because of uncertain factors, such as risk for movement/registration artifacts, suboptimal bolus with truncation of the arterial or venous curve due to the patient’s cardiac conditions, or suboptimal arterial input or venous output detection. Radiologists should be able to recognize these factors influencing the final results. Furthermore, post-processing algorithms and thresholding for core and penumbra volumes calculated from these relative perfusion maps over multiple vendors have no standardization [3].

In recent research [4], stroke patients with a large estimated core infarct on the non-enhanced head CT, with a low ASPECT score (3–5), or with an estimated ischemic core of 50 mL or greater due to a proximal large vessel occlusion showed better functional outcomes after endovascular thrombectomy compared to standard medical care. And regarding distal medium vessel occlusions (DMVOs), there is considerable interest [5]. Several multicenter trials are ongoing to show the efficacy and safety of treating these lesions with endovascular thrombectomy compared to standard medical care. These distal arterial occlusions (middle cerebral artery M2–M4, anterior cerebral artery, and posterior cerebral artery occlusions) are often difficult to identify on classical CT angiography (CTA) or MR angiography (MRA). Perfusion imaging is very beneficial in detecting these distal clots and decision-making.

For these reasons, one can assume that the number of perfusion studies, consequently CTP studies, will further increase. And this is where the shoe pinches for patients and radiologists. To ensure the identification of every acute ischemic stroke patient, CTP is progressively used as a screening tool, despite its association with higher radiation doses. But, shouldn’t we only accept low (or no) radiation dose screening tools? Radiologists are responsible for the radiation dose given to patients, the results, and the imaging interpretation. These arguments suggest that CTP is not an optimal screening tool for the detection and interpretation of ischemic stroke.

We should actively put more effort into gaining more information from non-contrast brain CT and multiphase CT angiography (mCTa). A recent publication showed a high accuracy of an AI model for non-contrast head CT for early stroke detection and estimation of stroke volumes, exceeding that of human experts, and approaching the ground truth accuracy of DWI on MRI [6]. This concept facilitates an interpretation of the core ischemic infarct from the non-enhanced CT of the brain, which is part of the standard-of-care imaging technique for every stroke patient.

A CT angiogram of the neck vessels and circle of Willis can be easily extended with late phases over the brain to obtain temporal information on the intracranial and, more specifically, pial arterial circulation [7]. The evaluation of mCTa is less complex and accurately provides the length of the intra-arterial clot [8]. More recent research also shows the potential to gain basic perfusion data, cerebral blood flow (CBF), and volume (CBV) from this temporal data [9]. These results might be obtained without the radiation burden from a CTP and with the help of AI models. But more research and clinical trials are needed in this particular field. Another direction is to focus on MRI availability. Are we able to optimize the installed base of MRI devices or do we have to focus on portable MRI devices [10]?

I want to raise awareness about the use of CTP techniques as a screening tool for detecting and evaluating ischemic volumes in stroke patients. MR(P) is, at this moment, the most accurate and safe technique from a radiation dose point of view to obtain this data. However, we must admit that, because of availability issues, CTP is the most used perfusion technique to evaluate acute stroke patients. As radiologists, we are responsible for the radiation dose to our patients and the interpretation of perfusion data, whether post-processed by the radiologist or completely automated software. Therefore, we should rethink the use of CTP for screening acute stroke patients.

## Declarations

### Guarantor

The scientific guarantor of this publication is Koenraad Nieboer MD.

### Statistics and biometry

No complex statistical methods were necessary for this paper.

### Methodology

• commentary
